# Patterns of Alcohol Consumption among Male Adults at a Slum in Kolkata, India

**DOI:** 10.3329/jhpn.v30i1.11279

**Published:** 2012-03

**Authors:** Santanu Ghosh, Amrita Samanta, Shuvankar Mukherjee

**Affiliations:** ^1^Department of Community Medicine, Calcutta National Medical College & Hospital, Kolkata, West Bengal, India; ^2^Department of Community Medicine, NRS Medical College & Hospital, Kolkata, West Bengal, India

**Keywords:** Alcohol, Alcohol consumption, Alcohol dependence, Community-based studies, Crosssectional studies, Harmful drinking, Hazardous drinking, India

## Abstract

Globally, alcohol-abuse is a major cause of mortality and morbidity. Consumption of alcohol has increased in India in the recent decades. It is imperative to know the patterns of alcohol consumption among different types of consumers to launch a well-planned nationwide programme for the prevention and control of this devastating social pathology. This community-based, cross-sectional study was undertaken to identify the patterns of alcohol intake among different types of alcohol consumers and to assess the clinical signs of chronic harmful alcohol-use. A predesigned, pretested, semi-structured alcohol-use disorders identification test (AUDIT) questionnaire was used for interviewing males, aged >18 years, selected by random sampling from an updated household list of a randomly-selected sector of the service area of the Urban Health Centre in Chetla, Kolkata, West Bengal, India. Written informed consents were obtained from all the respondents. Relevant clinical examination for chronic harmful alcohol-use was done according to the AUDIT clinical screening procedures. The results revealed that 65.8% (150/228) were current consumers of alcohol; 14% were alcohol-dependents; 8% were hazardous or harmful consumers, and 78% were non-hazardous non-harmful consumers. The mean age of the respondents at the initiation of drinking alcohol was 20.8+5.9 years. Eighty-six percent of dependents (n=21) took both Indian-made foreign liquor and locally-made alcoholic beverages. The proportions of alcohol consumers who drank alone among alcohol-dependents, hazardous or harmful consumers, and non-hazardous non-harmful consumers were 71.4%, 50%, and 7.7% respectively, and the difference was significant (p<0.01). Forty-one percent of the consumers drank at public places and workplaces, which may be socially harmful. About 38% of the dependents purchased alcohol from unlicensed liquor shops. Only 16% expressed concerns for their drinking habit mainly to the past illness. The proportion of the concerned respondents was higher in the hazardous and harmful drinking patterns than in the non-hazardous non-harmful drinking pattern, and the difference was significant (p<0.05). About 62% of the dependents had clinical signs of chronic alcohol consumption. The presence of a considerable proportion of alcohol-dependents, the low mean age at initiation of drinking alcohol, and the habit of drinking in public places and workplaces are the main areas that need special emphasis by intervention programmes.

## INTRODUCTION

Alcohol-abuse and alcoholism are one of the major public-health problems in both developed and developing countries (1). The 32nd World Health Assembly declared that “problems related to alcohol and particularly to its excessive consumption rank among the world's major public health problems and constitute serious hazards for human health, welfare and life” (2). The World Health Organization (WHO) estimated that there are about two billion consumers of alcoholic beverages and 76.3 million people with diagnosable alcohol-use disorders worldwide (3). Alcohol consumption accounts for nearly 3.2% of all days and 4% of all disability-adjusted life-years (DALYs) lost (4). In addition to chronic diseases, such as cancer of the mouth, oesophagus and larynx, liver cirrhosis, and pancreatitis, social consequences, such as road-traffic accidents, workplace-related problems, family and domestic problems, and interpersonal violence, have been receiving more public or research attention in recent years (4). The risks relating to alcohol are linked to the pattern of drinking and the amount of consumption. Many forms of excessive drinking cause substantial risk or harm to the individual. These include high-level drinking each day, repeated episodes of drinking to intoxication, and drinking that makes a person alcohol-dependent. While persons with dependence on alcohol are most likely to incur high levels of harm, the bulk of harms associated with consumption of alcohol occurs among non-dependent drinkers because they are more in number than dependents (5). Therefore, the identification of drinkers with various types and degrees of at-risk alcohol consumption has a great potential to reduce all types of alcohol-related harms (5).

Abuse of alcohol is one of the main killers of young men in India today. The mean age of respondents at the initiation of alcohol consumption has decreased from 23.36 years in 1950-1960 to 19.45 years in 1980-1990 (6). Due to its large population, India has become the third largest market for alcoholic beverages in the world. Despite having a large proportion of lifetime abstainers (89.6%), per-capita consumption of alcohol in India has increased by 106.7% over 1970-1996 (6). Changing social norms, urbanization, increased availability, high-intensity mass marketing, and relaxation of overseas trade rules, along with the poor level of awareness, have contributed to increased alcohol-use (6,7).

Growing urbanization is considered to play a pivotal role in the establishment and growth of slums (8). In India, 30-50% of the population of million-plus cities resides in slums. In Kolkata, about 7% of the total land area is occupied by slum-dwellers who constitute about 35% (1.5 million) of the total population residing in about 5,500 slums (2001) (9). Due to their deplorable socioeconomic status, slum-dwellers often remain the worst victims of the physical, psychological and social consequences of alcohol-use.

However, few studies have been conducted on the sociodemographic aspects and social consequences of alcohol-use among urban slum-dwellers in the eastern region of India, especially in West Bengal, despite the presence of a huge slum population in West Bengal, second only to Maharashtra in the country (8). A well-planned nationwide programme for the prevention and control of this social pathology is needed. The present study was undertaken with the objectives to identify the patterns of alcohol intake among different types of alcohol consumers and to assess the clinical signs of chronic harmful alcohol-use so that it might be beneficial in planning, implementation, and evaluation of appropriate programmes for the elimination of this social evil.

## MATERIALS AND METHODS

### Study setting and design

This community-based, cross-sectional study was conducted for one year (May 2008–April 2009) in the Sector IV of Unit B of the service area of the Urban Health Centre (UHC) in Chetla, the urban field practice area of the All India Institute of Hygiene and Public Health, Kolkata, West Bengal. The area is situated in the Chetla slum. There are four units (A, B, C, and D) in the service area of the UHC, and there are eight sectors (I to VIII) in these four units each containing two sectors within it. From these eight sectors, sector IV was randomly selected. An interview schedule was prepared following literature review and after consulting experts in the field of public health. The schedule was translated in Bangla as Bangla is the local language and was re-translated into English before its use. The schedule contained the alcohol-use disorders identification test (AUDIT) questionnaire, developed to screen for excessive drinking. Besides the AUDIT questionnaire, the schedule also contained questions relating to age at initiation of drinking alcohol, duration of current pattern of drinking, type of alcoholic beverages consumed, drinking companion, the most common time of drinking in a day, the most common place of drinking, the most common source of alcoholic beverages, concerns about drinking, and, if present, the most common reason for concerns. Results of the pilot study, conducted in the nearby Sector II among 40 adult males and 40 adult females, revealed that the prevalence of alcohol intake was <1% among the adult female population. Those males who were aged 18 years and above and those who voluntarily consented to participate were included as the study subjects.

Data of the pilot study showed that the prevalence of current alcohol consumers was 66%. Taking 10% allowable error at 95% confidence limit, the formula used for the estimation of sample-size was 4 pq/l^2^, where p is the prevalence of current consumers of alcohol; q is the prevalence of current non-consumers of alcohol (1-p); and l is 10% allowable error. The estimated sample-size was 206. Taking 10% non-response rate, the final sample-size estimated was 227.

According to the records available at the UHC, the total number of adult males aged 18 years and above in the study area was 1,440. A list of houses obtained from the UHC was verified and updated by the researchers before conducting the actual study. The study subjects were then selected by simple random sampling from the updated list of the residents of the area.

### Collection and analysis of data

The study participants were interviewed using a predesigned, pretested, semi-structured schedule. Relevant clinical examination of each current alcohol consumer was done according to the AUDIT clinical screening procedures, depicting the common signs of chronic harmful alcohol-use, namely conjunctival injection, abnormal skin vascularization, hand tremor, tongue tremor, and hepatomegaly. The non-response rate was <1%. The number of the final study subjects was 228. Of them, 150 were current alcohol consumers who were considered subjects in the present study. Finally, data were collected and analyzed using the frequency distribution tables and proportions.

### Definitions used in the study

**Current drinker:** Current drinkers included those who consumed one or more drinks of any type of alcohol in the year preceding the survey (10).

**Hazardous drinking:** It is a pattern of alcohol consumption that increases the risk of harmful consequences for the user or others. The hazardous drinking patterns are of public-health significance, despite the absence of any current disorder in the individual user (5).

**Harmful use:** It refers to the consumption pattern of alcohol that results in physical and mental health consequences, including guilt-feeling after drinking, blackouts, alcohol-related injuries, and concern of other people about drinking (5).

**Alcohol dependence**: It is a cluster of behavioural, cognitive and physiological phenomena that may develop after repeated alcohol-use. Typically, these phenomena include a strong desire to consume alcohol, impaired control over its use, persistent drinking despite the harmful consequences, a higher priority given to drinking than to other activities and obligations, increased alcohol tolerance, and a physical withdrawal reaction when alcohol-use is discontinued (5).

**Former drinker:** The former drinkers include those who have ever drunk alcohol but those who did not consume 1 or more drinks in the year preceding the survey (10).

**Lifetime abstainer:** The lifetime abstainers include those who never consumed 1 or more drinks of any type of alcohol (10).

**One standard drink:** One standard drink includes: 1 can beer (330 mL) at 5%x(strength) x 0.79 (conversion factor)=13 g of ethanol or 1 glass of wine (140 mL) at 12% x 0.79=13.3 g of ethanol or 1 shot spirits (40 mL) at 40% x 0.79=12.6 g of ethanol (5).

In the case of the respondents who consumed locally-made liquor, one drink was measured locally by observing the proportion of alcohol written on the label of the liquor bottle and then by asking him to show the content in a container of known volume carried by the researcher.

### AUDIT questionnaire

The AUDIT questionnaire is the only screening test specifically designed for international use (5). The questionnaire helps the practitioner identify whether the person has hazardous drinking, harmful drinking, or alcohol dependence. It is a 10-item questionnaire (Annexure); it takes about two minutes to complete; each question is scored from 0 to 4; and the maximum score is 40. A total score of 8 or more on the questionnaire suggests that the person has a pattern of hazardous or harmful alcohol consumption. As a general guide, if a score is 13 or more, it is likely that the person is alcohol-dependent. Those having a total score of less than 8 were called ‘non-hazardous non-harmful’ by the researchers due to lack of a suitable nomenclature in literature regarding the same.

### Statistical analysis

Statistical analysis was performed by proportion, mean, and chi-square test. The Microsoft Excel Office 2007 and the Epi Info software (version 3.3.2) were used for statistical analysis.

### Ethical approval

Written informed consent was obtained from each respondent, ensuring strict anonymity. The Ethical Committee of the All India Institute of Hygiene and Public Health approved the study.

## RESULTS

Of the 228 respondents, 65.8% (n=150) were current alcohol consumers. Of the non-consumers (n=78), 5.3% were former drinkers, and 28.9% were lifetime abstainers. According to the AUDIT score, 78% of the current alcohol consumers had a non-hazardous non-harmful pattern, 8% had a hazardous or harmful pattern, and 14% had alcohol dependence. The mean age of the consumers was 31.4±10.8 years. Of the alcohol-dependents, 50% were aged 50-59 years, and 17.6% were aged 30-39 years. All the hazardous or harmful consumers were aged 20-39 years. The mean age of the alcohol-dependents was more (38.7±13.9) than that of the non-hazardous non-harmful and hazardous or harmful consumers (30.5±10.8 and 28.8±5.7 respectively). The mean age of the respondents at the initiation of alcohol consumption was 20.5±5.7 years, and the mean duration of drinking was 6.7±5.8 years. The mean age at initiation and the mean duration of drinking among the consumers belonging to all the three patterns were almost similar. The mean ages at which the non-hazardous non-harmful consumers, hazardous or harmful consumers, and alcohol-dependents started drinking alcoholic beverages were 20.9±6.4, 19.4±1.0, and 19.3±1.7 years respectively. The mean durations of drinking from their initiation up to the time of study were 6.5±4.9, 5.3±1.7, and 7.6±10.2 years among the non-hazardous non-harmful consumers, hazardous or harmful consumers, and alcohol-dependents respectively. The duration of drinking among 39.3% of the consumers was 1-4 years, followed by 5-9 years (38.0%). However, no definite relationship was observed between the duration and the pattern of consumption. Regarding the type of liquor most commonly consumed, about 51% consumed only Indian-made foreign liquor, 14.7% consumed locally-made liquor, and 34% consumed both the types of liquor. It was also observed that 85.7% of the respondents having alcohol dependence and 66.7% of those having hazardous or harmful consumption took both the types of liquor. No alcohol-dependents took only foreign liquor, and no hazardous or harmful consumers took only locally-made liquor. Eighty percent of the consumers drank usually with others. The proportions of consumers who drank alone among alcohol-dependents, hazardous or harmful consumers, and non-hazardous non-harmful consumers were 71.4%, 50%, and 7.7% respectively, and the difference was significant (p<0.01). Most (91.3%) consumers consumed alcohol in the evening and night. All the alcohol-dependents and hazardous or harmful consumers consumed alcohol in the evening and night. Most (90%) consumers preferred to drink outside the home. About 41% of them used to drink at the public places and workplaces, and 18% preferred the home of their friends as the most common place of drinking whereas 28.6% of the alcohol-dependents preferred the public places or liquor shops (28.6% each). Fifty percent of the hazardous or harmful consumers preferred the workplace for drinking. Most (94.7%) consumers purchased alcohol from licensed liquor shops, and most (80%) of these liquor shops (n=5) were located near their home. Of them, about 6% purchased alcoholic beverages from bars. However, 5.3% of the study subjects and 38.1% of the alcohol-dependents purchased alcoholic beverages from local unlicensed liquor shops (Table 1).

It was observed that 76.7% of the consumers had no clinical sign of chronic harmful alcohol-use. However, 18% had hand tremor, 10% had tongue tremor, and 10% had conjunctival injection. All the signs were found more among alcohol-dependents compared to other two categories. All the alcohol-dependents had at least one of the signs. About 62% of the alcohol-dependents, 3.9% of the non-hazardous non-harmful consumers, and 33.3% of the hazardous and harmful consumers had conjunctival injection. About 24% of the dependents had scleral jaundice whereas none of the consumers belonging to other two categories had this sign. Of the alcolol-dependents, 85.7% had hand tremor whereas it was found in 41.7% of the hazardous or harmful consumers. Tongue tremor was found in 80.9% of the alcohol-dependents and in 33.3% of the hazardous or harmful consumers. Hepatomegaly was found in 14.3% of the alcohol-dependents and in 1.3% of the non-hazardous non-harmful consumers (Table 1).

The results revealed that 84% of the consumers were not concerned for their consumption of alcohol. Only 24 (16%) respondents expressed concern for their drinking habits. About 71% of the alcohol-dependents expressed no concerns for their drinking habits. The proportions of the respondents who were concerned for their drinking habits was higher among the hazardous or harmful consumers than among the non-hazardous non-harmful consumers, and this difference was significant (p<0.05). Only 25% of the consumers expressed their concerns either to the past history of illness or to the fear of developing physical illness (25% each). Increases in expenditure because of drinking and bad influence on children were also among the important causes of concern (16.7% each). Restrictions imposed by relatives (12.5%) also played an important role as a cause of concern (Table 2).

## DISCUSSION

The problem of alcohol consumption in India has widely attracted the attention of the public, policy-makers, researchers, and workers. Taxes from alcohol production and sales are the major sources of revenue (Rs 25,000 crore) in most states and have been cited as a reason for permitting the sale of alcohol (11). However, closer inspection revealed that out-of-pocket expenses (Rs 244 million) consequent to alcohol consumption was higher than the total excise revenue from alcohol (Rs 214 million) in 2004 (12). During the past 35 years, several field surveys of general psychiatric morbidity and alcohol consumption have been carried out in different parts of the country. The overall prevalence of alcohol intake was estimated to be 6.9 per 1,000 people with urban preponderance (7.3 per 1,000 vs 5.8 per 1,000 in rural area) (13). The present study reported the prevalence of 65.8% (150 per 228 people).

**Table 1.** Different sociodemographic correlates of patterns of drinking (n=150)

Sociodemographic characteristics	Non-hazardous non-harmful (n=117)	Hazardous or harmful (n=12)	Alcohol dependent (n=21)	χ^2^ value, df, p value
Age (years) at initiation				
<20 (n=69)	57 (82.6)	3 (4.3)	9 (13.1)	2.56, df: 2, p=0.28
≤20 (n=81)	60 (74.1)	9(11.1)	12 (14.8)	
Duration (years) of drinking				
<5 (n=62)	47 (75.8)	3 (4.8)	12 (19.4)	3.55, df: 2, p=0.17
≤5 (n=88)	70 (79.6)	9 (10.2)	9 (10.2)	
Type of liquor				
Only foreign liquor (n=77)	73 (94.8)	3 (3.9)	1 (1.3)	35.5*, df: 4, p=0.00
Only locally-made liquor (n=22)	18 (81.8)	1 (4.5)	3 (13.7)	
Both (n=51)	26 (51.0)	8 (15.7)	17 (33.3)	
Drinking companion				
Alone (n=30)	9 (30.0)	6 (20.0)	15 (50.0)	52.5*, df: 2, p=0.00
With others (n=120)	108 (90.0)	6 (5.0)	6 (5.0)	
Most common places of drinking				
Home (own/friends) (n=42)	36 (85.8)	3 (7.1)	3 (7.1)	3.28, df: 4, p=0.51
Workplaces and public places* (n=62)	47 (75.8)	6 (9.7)	9 (14.5)	
Liquor shops and others† (n=46)	34 (40.0)	3 (20.0)	9 (40.0)	
Most common time of drinking				
Morning and afternoon (n=13)	13 (8.7)	0	0	
Evening and night (n=137)	104 (69.3)	12 (100.0)	21 (100.0)	
Most common source of purchase of alcohol				
Licensed liquour shop (n=142)	117 (100.0)	12 (100.0)	13 (61.9)	
Unlicensed liquor shop	0	0	8 (38.1)	
Whether concerned for drinking habit				
No (n=126)	108 (92.3)	3 (33.3)	15 (71.4)	35.7*, df: 2, p=0.00
Yes (n=24)	9 (7.7)	9 (66.7)	6 (28.6)	
Clinical findings for chronic harmful alcohol consumption‡				
Conjunctival injection	3 (3.9)	4 (33.3)	13 (61.9)	20 (13.3)
Scleral jaundice	0	0	5 (23.8)	5 (3.3)
Hand tremor	3 (3.9)	5 (41.7)	18 (85.7)	26 (17.3)
Tongue tremor	2 (2.6)	4 (33.3)	17 (80.9)	23 (10.0)
Hepatomegaly	1 (1.3)	0	3 (14.3)	4 (2.7)

*Public places included local ground and common premises;

†Others included homes of relatives, office superior, etc.;

‡Multiple responses;

df=Degree of freedom

**Table 2.** Most important cause of concern for alcohol consumption (n=24)

Cause of concern	No.	%
Past history of physical illness	6	25.0
Fear of developing physical illness	6	25.0
Bad influence on children	4	16.7
Increase in family expenses	4	16.7
Disliked by relatives	3	12.5
Getting old	1	4.1

Girish reported the prevalence of 67.4% among the 26-45-year age-group (14). Singh *et al*. reported that 87.5% of urban males consumed alcohol daily in Amritsar, Punjab, which is much higher than that in the present study (15). According to the AUDIT score, 78% had the non-hazardous non-harmful pattern, 8% had the hazardous or harmful pattern, and 14% had alcohol dependence among the current alcohol consumers in the present study. Dhupdale *et al*. reported the prevalence of 9% and 1% hazardous or harmful consumers and alcohol-dependents respectively according to the AUDIT score in the age-group of 19 years and above (16), which is lower than that in the present study. The results of the present study showed that the mean age of the consumers at the initiation of consuming alcoholic beverages was 20.5±5.7 years, which is considerably low. Overall, the age-range at initiation of drinking was 20-29 years as found in different studies, despite the wide differences among regions, populations, and years of studies (17-21), similar to that in the present study. The National Family Health Survey (NFHS) 3 revealed the 13.4% prevalence of alcohol intake among the 15-49 years age-group (22).

In the present study, it was found that about 51% took only Indian-made foreign liquor, 14.7% took only locally-made alcoholic beverages, and 34% took both. About 5% of the drinkers consumed illicit alcoholic beverages. This finding corroborates the finding of Benegal *et al*. who observed that the most common beverage used by men was Indian-made foreign liquor (70.7%) (23); only 27% restricted themselves to only one type of beverage, and 7.7% used illicit alcoholic beverages (24). Girish also reported that whisky was the most preferred type of liquor in urban areas (63%) (14). In contrast, Deswal observed that 53.3% of alcohol consumers in Arunachal Pradesh consumed local home-made beverages (17). The difference in the type of beverages across studies in cities and those in remote areas might be due to the fact that foreign liquors were more easily available at a relatively-lower cost in a mega city like Kolkata or Bangalore than in a hilly area of Arunachal Pradesh, and beverage-making with local grains by families was very popular and a prevalent practice in the area.

In the present study, 80% of the consumers drank usually with others. This finding is similar to that of Deswal (17). In the present study, most (91.3%) consumers consumed alcohol in the evening and night. Regarding the place of drinking, most (90%) consumers preferred to drink outside the home. About 41% of them used to drink at the public places and workplaces. About 18% preferred the home of their friends as the most common place of drinking. Public places (28.6%) and liquor shops (28.6%) were the most preferred places of drinking among the alcohol-dependents. Fifty percent of the hazardous or harmful consumers preferred the workplaces for drinking. Neogi *et al*. observed that 75.7% of regular alcohol drinkers preferred to consume alcoholic beverages with their relatives and friends (18). All the alcohol-dependents and hazardous or harmful consumers consumed alcohol in the evening and night. This finding corroborates the finding of Benegal *et al*. who observed that the most commonly-reported time of drinking of 92% of drinkers was the weekend after 5 pm. About 20% of males reported drinking everyday or almost every day in the evening (23).

### Limitations

The most important limitation is that the present study was cross-sectional in design; thus, there was no scope for follow-up of the study subjects for any change in the pattern of drinking habits and clinical signs. More analytical studies, especially of longitudinal design, are required to establish the association of different sociodemographic variables with alcohol consumption and consistency of different patterns of consumers. Another limitation was that the study was conducted in a single slum, and the findings may not be applicable to other slum populations of Kolkata. The possibility of conscious falsification on the sensitive issues could not be ruled out, despite the sincere efforts by the researchers regarding confidentiality. The advantages of cross-sectional design are that the study was easy to conduct, relatively inexpensive, and easy to get cooperation from participants because data are collected only once.

### Conclusions

The presence of a considerable proportion of alcohol-dependents, the low mean age at initiation of drinking, and the habit of drinking in the public places and workplaces remained the main areas to be emphasized for the successful implementation of intervention programmes. The intervention programmes should include preventive strategies and screening programmes to identify different patterns of drinkers and treatment intervention for alcohol-dependents. The preventive strategies should include reduction of access to alcohol by formulating and enforcing laws regarding alcohol consumption. First of all, a comprehensive Central Government act like ‘The Cigarettes and Other Tobacco Products Act, 2003’ (24), encompassing the prohibition of advertisements, regulation of trade and commerce, and production, supply, and distribution of alcoholic beverages, must be urgently passed and implemented as an initial step to regulate the accessibility of people to alcohol. The legal age of drinking must be clearly delineated, and prohibitive measures must be properly implemented. Again, legal prohibition on drinking in the public places and workplaces must be strictly enforced. A number of factors play a role in the physical availability of alcoholic beverages, including the times of sales permitted, types, characteristics, and location of outlets, and the distribution system of alcoholic beverages. Specific licensing of alcohol outlets, limits on the number of outlets, and on times and conditions of alcoholic beverage sales or service are to be implemented. The results of the present study revealed that most consumers drank in the evening. Legal measures to close the liquor shops at definite hours are to be implemented and sustained. Training programmes of local health workers regarding the abuse of alcohol and other substances and their health and social consequences must be conducted at regular intervals.

## ACKNOWLEDGEMENTS

The authors are grateful to Dr. Ranadeb Biswas, Professor and Head and Dr. Aparajita Dasgupta, Professor, Department of Preventive and Social Medicine, All India institute of Hygiene and Public health, Kolkata, for providing valuable suggestions and guidance; Dr. Sushil Sen, Public Health Specialist, Urban Health Centre, Chetla, for his constant encouragement; Public Health Nurses, Urban Health Centre, Chetla, to introduce the authors to the people of the study area; and last but not the least, residents of the Sector IV in Chetla, without whom the study would not have been possible.

## Annexure. Alcohol intake disorders identification test

Please circle the answer that is correct for you

1. How often do you have a drink containing alcohol?
  □ Never □ Monthly or less □ 2-4 times a month □ 2-3 times a week □ 4 or more times a week
2. How many drinks containing alcohol do you have on a typical day when you are drinking?
  □ 1 or 2 □ 3 or 4 □ 5 or 6 □ 7 to 9 □ 10 or more
3. How often do you have six or more drinks on one occasion?
  □ Never □ Less than monthly □ Monthly □ Weekly □ Daily or almost daily
4. How often during the last year have you found it difficult to get the thought of alcohol out of your mind?
  □ Never □ Less than monthly □ Monthly □ Weekly □ Daily or almost daily
5. How often during the last year have you found that you were not able to stop drinking once you had started?
  □ Never □ Less than monthly □ Monthly □ Weekly □ Daily or almost daily
6. How often during the last year have you been unable to remember what happened the night before because you had been drinking?
  □ Never □ Less than monthly □ Monthly □ Weekly □ Daily or almost daily
7. How often during the last year have you needed a first drink in the morning to get yourself going after a heavy drinking session?
  □ Never □ Less than monthly □ Monthly □ Weekly □ Daily or almost daily
8. How often during the last year have you had a feeling of guilt or remorse after drinking?
  □ Never □ Monthly □ Weekly □ Daily or almost daily
9. Have you or someone else been injured as a result of your drinking?
  □ No □ Yes, but not in the last year □ Yes, during the last year
10. Has a relative, friend, doctor, or any other health worker been concerned about your drinking or suggested you cut down?
  □ No □ Yes, but not in the last year □ Yes, during the last year
